# Predictors of Nursing Home Entry within 36 Months after Hospitalization via the Emergency Department among Persons Aged 75 Years or Older

**DOI:** 10.3390/geriatrics8030067

**Published:** 2023-06-15

**Authors:** Moustapha Dramé, Alison Volberg, Lukshe Kanagaratnam, Claire Coutureau, Lidvine Godaert

**Affiliations:** 1 EpiCliV Research Unit, Faculty of Medicine, University of the French West Indies, Fort-de-France F-97200, Martinique; 2 Department of Clinical Research and Innovation, University Hospitals of Martinique, Fort-de-France F-97200, Martinique; 3 Department of Clinical Research and Innovation, University Hospitals of Reims, F-51100 Reims, France; 4 Department of Geriatrics, General Hospital of Valenciennes, F-59300 Valenciennes, France

**Keywords:** nursing home entry, older adults, emergency department, prediction

## Abstract

Objective: We aimed to identify risk factors for nursing home (NH) entry 36 months after hospitalization via the emergency department (ED) in a population of patients aged 75 years or older. Methods: This was a prospective multicentre cohort. Patients were recruited from the emergency departments (EDs) of nine hospitals. Subjects had been hospitalised in a medical ward in the same hospital as the ED to which they were initially admitted. Subjects who experienced NH entry prior to ED admission were excluded. NH entry has been defined as the incident admission either into an NH or other long-term care facility within the follow-up period. Variables from a comprehensive geriatric assessment of patients were entered into a Cox model with competing risks to predict NH entry during 3 years of follow-up. Results: Among 1306 patients included in the SAFES cohort, 218 (16.7%) who were already in an NH were excluded. The remaining 1088 patients included in the analysis were aged 84 ± 6 years on average. During 3 years of follow-up, 340 (31.3%) entered an NH. The independent risk factors for NH entry were that they: living alone (Hazard ratio (HR) 2.00, had a 95% confidence interval (CI) 1.59–2.54, *p* < 0.0001), could not independently perform activities of daily living (HR 1.81, 95% CI 1.24–2.64, *p* = 0.002), and had balance disorders (HR 1.37, 95% CI 1.09–1.73, *p* = 0.007), dementia syndrome (HR 1.80, 95% CI 1.42–2.29, *p* < 0.0001) and a risk of pressure ulcers (HR 1.42, 95% CI 1.10–1.82, *p* = 0.006). Conclusion: The majority of the risk factors for NH entry within 3 years after emergency hospitalization are amenable to intervention strategies. It is therefore reasonable to imagine that targeting these features of frailty could delay or prevent NH entry and improve the quality of life of these individuals before and after NH entry.

## 1. Introduction

The part of the general population aged 65 or over, totalling 13.1 million in 2018 in France, has risen steadily in recent years from 15.5% in 1998 to 19.6% in 2018. In high-income countries, the progressive increase in the population in the next few years will be mainly concentrated in the 65 or over age group. While less than 2% of 65 to 74 years old lived in institutions in 2015, the proportion of those aged 85 or over reached 21%. In France, the average age of entry into an institution was 86 years and one month [1].

Nursing home (NH) admission is a frequent source of concern for older persons, their caregivers, and also for societies that contribute to financing NHs [2]. With the anticipated aging of the population, this question will become increasingly pressing over the coming decades, and so too will high levels of dependency during their last years of life. With the anticipated aging of the population, this question will become increasingly pressing over the coming decades. This will also be the case of dependency during their last years of life. There is a general consensus that the later NH entry occurs, the better the outcome will be, not only for economic reasons [3], but also because the majority of aging adults prefer to “age in place”. Campbell-Enns et al. [4] reported that NH entry is the last resort for socially isolated older adults who are losing autonomy. A number of non-modifiable risk factors for NH entry have been identified, and the risk is greater when these factors accumulate [5,6], e.g., being widowed [5], living alone [6,7,8], and having a cognitive impairment [5,6,9], multimorbidity or Parkinson’s disease [6]. Advancing age is another non-modifiable risk factor for admission to an NH [6,8]. While some of these factors are associated with the short-term need for NH entry [10], other factors may be amenable to early interventions that could mitigate their impact, and thus delay NH entry, such as gait disorders [5,9,10,11]. There is, therefore, a need for the early identification of factors associated with the need for NH admission among older adults, with a view to target them for intervention, ultimately, delaying or preventing NH entry. In this context, the aim of this study was to identify the risk factors for NH entry in a population of adults aged 75 years or older who were hospitalized via emergency departments (ED).

## 2. Methods

### 2.1. Study Design

The SAFES study is a prospective, multicentre cohort, which was set up in the framework of a national hospital-based clinical research programme (PHRC), with recruitment from the EDs of nine French hospitals (eight university hospitals and one regional (non-academic) hospital). Each of these nine hospitals has a geriatric short-stay unit. The methods of the SAFES study have previously been described elsewhere [12].

### 2.2. Study Population

Inclusion ran over a period of 11 months (from February 2001 to January 2002). To be eligible, patients had to be aged 75 years or over and have been admitted to a medical ward in the hospital after attending the ED of that same hospital. Patients were not eligible if they had been admitted to the intensive care unit (ICU) or had undergone surgery in the ED, or if admission did not occur after admission to the ED. Every day in each centre, patients admitted to the emergency unit were registered. From the list thus obtained, patients were selected via a random draw that was stratified at two levels: in each week, 5 days were selected randomly, and for each of these days, two patients were chosen randomly. Each patient was visited by a specialist in geriatrics who was familiar with the survey procedures. During the course of this interview, patients were informed about the study prior to signing the consent form. If the clinical status and/or the cognitive status of the patient did not enable them to give informed consent, the interviewer referred to the subject’s representative. A follow-up was performed via face-to-face interviews at inclusion, at 5, 12, 18 and 24 months and via telephone interviews at 1, 8, 21 and 36 months after index hospitalisation. NH entry has been defined as the incident admission either into an NH or other long-term care facility within the follow-up period. Patients who had been admitted to a nursing home prior to admission to the emergency department (ED) were excluded from the analyses. There was no formal sample size calculation. It was pragmatically decided that each centre could include an average of 10 to 15 patients per month over a twelve-month period.

### 2.3. Variables Studied

Socio-demographic and clinical information were collected. The sociodemographic variables included age, gender, education level and social environment. A comprehensive geriatric assessment (CGA) was performed. The level of independence in performing basic activities of daily living (ADL) was assessed using the Katz scale [13]. The patient was considered to be dependent if he/she was unable to perform at least one of the following six activities: bathing, dressing, toileting, transferring, feeding or continence. Mood disorders and the risk of depression were assessed using the Schwab depression scale, altered by Gilleard, and defined as a score >14 [14]. Dementia and delirium were defined according to the DSM-IV criteria [15]. Nutritional status was assessed via the Mini Nutritional Assessment [16] and via obtaining serum albumin levels. An MNA score <24 or a serum albumin level <35 g/L was defined as undernutrition. Walking and fall risk were assessed via the timed get-up and go test [17] and the one-leg balance test [18], respectively. A timed get-up and go test >20 s indicated that the patient experienced walking difficulties. A one-leg balance of <5 s on either leg indicated that the patient was at fall risk. An ICD10-adapted version of the Charlson index [19] was used to estimate the level of comorbidity. The risk of pressure ulcer was assessed using the Norton scale, and a score of ≤14 indicated a risk of developing a pressure ulcer [20]. The presence of sensory problems (auditory or visual) despite the use of correction was noted. We also recorded any aids used (formal and informal aids). Finally, the conditions of admission to the ED were recorded, namely, the day of admission (weekday: from Monday to Friday vs. weekend) and whether or not they had been previously hospitalized within the preceding three months.

### 2.4. Ethical Considerations

This study was carried out in accordance with the Helsinki Declaration and current French legislation on biomedical research. It was approved by the national ethics committee responsible for ensuring the protection of people included in biomedical research. Patients were free to opt out of the study at any time by making a simple request, without any impact on the care provided.

### 2.5. Statistical Analyses

Continuous variables are described as means and standard deviations (m ± SD), and categorical variables are described as numbers and percentages (*n*, %). The dependent variable was time to NH entry over the 36 months of follow-up. The term “Nursing home” was defined as nursing homes or other any long-term care facilities. The start of the follow-up corresponded to the date of the arrival at the ED. Patients who were lost to the follow-up were censored at the date of their last contact. Bivariable and multivariable analyses were performed using Cox models, taking into account competing risks between death and NH entry [21]. For multivariable analysis, the Cox regression model was used in a stepwise method after looking for confounders and interactions. Variables with *p* < 0.20 according to bivariable analysis were proposed in the multivariable analysis. the removal threshold was *p* > 0.10. The hypotheses of log-linearity and the proportionality of risks were verified using the graphical method [22]. Analyses were performed with SAS version 9.4 (SAS Institute Inc., Cary, NC, USA), and the level of statistical significance was set at *p* < 0.05.

## 3. Results

**Figure 1:**
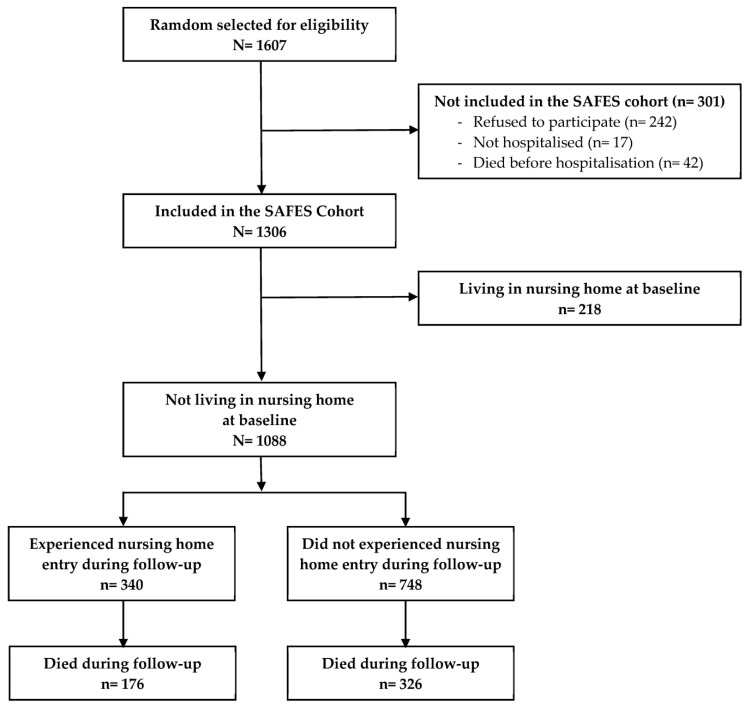
Flow diagram of the selection process.

Of the 1306 patients included in the SAFES cohort, 218 were excluded because they were already living in an NH at the baseline. The remaining 1088 patients were included in the final analysis (see Figure 1). The average age was 84 ± 6 years old. The mean Charlson comorbidity score was 1 ± 1. Half of the patients (49%) were living alone, one third (33%) did not have a main caregiver, and seventy percent had formal help. The other main baseline characteristics of the study population are presented in Table 1.

Figure 1 describes the selection process of the study population. During the 36 months of follow-up, 340 individuals (31.3%) entered an NH, and 326 (30.0%) died without being admitted to an NH (competing risk). Among the 340 who entered an NH, 176 (51.8%) died before the end of the 36 months of the follow-up. The overall mortality rates during the follow-up were 10.6%, 34.1%, 44.0% and 49.9% at 6 weeks, and 12, 24 and 36 months, respectively.

The results of the multivariable analysis are presented in Table 2. The independent risk factors for NH admission during the 36 months of the follow-up included social factors (living alone: OR = 2.00; 95% CI = 1.59–2.54; *p* < 0.0001), as well as medical factors, such as independence in performing ADLs (OR = 1.81; 95% CI = 1.24–2.64; *p* = 0.002), a fall risk (OR = 1.37; 95% CI = 1.09–1.73; *p* = 0.007), dementia syndrome (OR = 1.80; 95% CI = 1.42–2.29; *p* < 0.0001) and the risk of developing pressure ulcers (OR = 1.42; 95% CI = 1.10–1.82; *p* = 0.006).

## 4. Discussion

In a population of over 1000 multimorbid and dependent older individuals, our study identified five risk factors present at baseline and associated with NH entry within three years after hospitalization via the ED, namely, living alone, independence in performing ADLs, a fall risk indicated by having balance disorders, cognitive impairment, and the risk of developing pressure ulcers. These factors are congruent with those previously identified in the literature, notably, living alone [6,7,8,23], the presence of cognitive impairment [5,6,8] or walk disturbances and fall risk [5,9,10,11]. The risk factors identified in this study reflect the presence of multidimensional frailty in the three years prior to NH entry, including social, motor, functional and cognitive frailty, as well as nutrition problems. All these domains are interrelated and may concurrently deteriorate. Consequently, a secondary NH admission may result from the failure of ambulatory care to compensate for these impairments [4], or from deterioration in one or more of these domains, thereby requiring specialized and permanent assistance.

Nevertheless, some of the factors identified in this study are amenable to intervention, with a view to reinforcing the capacity of older persons. This is the case, for example, for social frailty (as reflected by living alone), functional frailty (the loss of ADLs), and motor and nutritional frailty (fall risk and risk of developing pressure ulcers). Indeed, living alone is a key component of social frailty [24,25]. Makizoka et al. previously showed that social frailty contributed to the loss of autonomy among older adults [26], even in those who are not physically frail [27]. Huang et al. also reported that social frailty exposed older adults to the risk of psychological and cognitive decline [28]. Despite the differences that may exist between countries, particularly in terms of the profile of the elderly population or in terms of the type of institution housing the elderly, social isolation is found, in several countries, to be a risk factor for entering an institution [29,30]. Implementing strategies to reduce social frailty seems to be an attractive, accessible and likely profitable option. Multiple strategies could be envisaged, such as increasing social interactions through social clubs for seniors, intergenerational housing, attendance at day-care centres, etc. The financial aspects and individuals’ mobility also need to be taken into account when aiming to reduce social frailty. Functional frailty, as reflected by loss of the ADLs, is very common among older adults. Its prevalence increases with age [31], affecting almost 70% of those aged 90 and more than 95% of those who live to be one hundred [32]. This form of frailty seems to be an inexorable effect of aging, given its exponentially increasing prevalence with increasing age [33]. Strategies to combat functional frailty combine the rehabilitation of older adults, the adaptation of their environment to meet their capacity and reinforced formal or informal assistance. Some of these approaches overlap with the possible strategies to mitigate social frailty, notably increased interactions in the home. The burden of functional frailty is heavy for the families and/or carers of affected older adults, and the caregiver burden is at the origin of a certain proportion of NH entries [4], highlighting the importance of professional aid. Motor and nutritional frailty are intricately linked, with undernutrition contributing to sarcopenia [34]. These two domains are amenable to strategies aimed at consolidating an individual’s personal capacity, and potential interventions could include walking rehabilitation, dietary supplementation with proteins, energy and vitamins, and also reinforced social contacts and cognitive stimulation. In a systematic review, Beaudart et al. [35] underlined the proven efficacy of physical exercise in combating sarcopenia, whereas isolated nutritional interventions have a limited impact. This emphasizes the importance of implementing multifaceted interventions targeting the multidimensional and interdependent nature of frailty domains. Since the opportunities for improving each individual domain of frailty are limited, it is desirable to attempt to durably and simultaneously consolidate several domains to achieve efficacious and lasting results. Pursuing the consolidating actions over the medium-to-long term is also of paramount importance.

Duan-Porter et al. [2] performed a systematic review of reviews in the literature investigating the preventive actions implemented to delay NH entry in a population of older adults. The mean age was not indicated, but 10 studies out of 47 specifically concerned older adults with or without cognitive impairment. These authors found that the majority of approaches targeted carers, and all the programmes were carried out in high-income countries. Overall, after their review, the authors did not find any noteworthy efficacy of this type of intervention. However, none of the studies included investigated interventions specifically focussed on consolidating identified frailty domains. Entry into an institution is sometimes the result of comorbidities. Marengoni et al. [36] compared six patterns of multimorbidity and the 6-year risk of NH admission among older people in Sweden. Three mutlimorbidity patterns were at increased risk: cardiovascular disease, anaemia and dementia. NH admission can be considered as an alternative to frequent hospitalisations or as a solution to an increased need for medical follow-ups. In this study, dementia was also identified as risk factor for entry into an institution.

This study has some limitations that deserve to be underlined. First, the study population is likely not representative of the overall general population of those aged 75 or over admitted to EDs, since we excluded those who were admitted to ICUs or who had undergone surgery. However, this exclusion was necessary, since CGA could not be performed in those patients. Second, our results were not externally validated. Nevertheless, the findings are in line with our working hypotheses and coherent with data in the literature. This study also has some strongpoints including the robust quality of the results thanks to multicentre inclusions, the large number of subjects, and high event rates, thereby conferring good statistical power onto the analyses. Second, candidate variables were taken from CGA performed by trained evaluators. Third, the statistical model used took into account the competing risk of death when judging the hazard of NH entry.

## 5. Conclusions

This study identified risk factors associated with NH entry within 36 months after hospitalization via EDs in a population of multimorbid, dependent subjects aged 75 years or older. The majority of these risk factors are amenable to interventions aimed at consolidating their capacity. It is, therefore, reasonable to postulate that consolidating these domains of frailty could help to delay NH entry, while improving the quality of life in this population, both before and after NH admission. Screening for these risk factors could be carried out regularly by first-line healthcare providers, ideally before hospital admission.

## Figures and Tables

**Table 1:** Baseline demographic and medical characteristics of the study population (N = 1088).

Characteristics	N	%
Age (years)		
<85	592	54.4
≥85	496	45.6
Sex		
Female	667	61.3
Male	421	38.7
Level of education		
Primary school (or less)	719	70.0
Secondary school	216	21.0
University	92	9.0
Living alone		
No	542	50.8
Yes	526	49.2
Main caregiver		
No	356	32.7
Yes	732	67.3
Formal help		
No	314	30.2
Yes	726	69.8
Informal help		
No	121	11.6
Yes	919	88.4
Dependence for the ADLs		
No	180	17.2
Yes	869	82.8
Walking difficulties		
No	225	20.7
Yes	862	79.3
Balance difficulties		
No	525	48.9
Yes	548	51.1
Dementia syndrome		
No	629	58.4
Yes	449	41.6
Delirium		
No	857	79.5
Yes	221	20.5
Depression or risk thereof		
No	641	58.9
Yes	447	41.1
Serum albumin (g/L)		
≥35	445	41.8
<35	633	58.2
Total MNA Score		
≥24	449	41.3
<24	639	58.7
Risk of pressure ulcers		
No	711	65.5
Yes	374	34.5
Visual impairment		
No	534	51.4
Yes	505	48.6
Hearing impairment		
No	613	56.6
Yes	470	43.4
Recent hospital admission		
No	781	73.1
Yes	288	26.9
Day of hospital admission		
Weekday	835	76.8
Weekend	253	23.2

**Table 2:** Factors associated with 36-month nursing home entry. Bivariable and multivariable analyses (N = 1088).

	Bivariable Analysis			Multivariable Analysis		
Characteristics	OR	IC 95%	*p*	OR	IC 95%	*p*
Age (years): ≥85	2.16	1.73–2.68	<0.0001			
Male sex	0.74	0.59–0.94	0.01			
Level of education						
Primary school (or less)	1					
Secondary school	0.96	0.73–1.27	0.79			
University	0.74	0.48–1.14	0.17			
Living alone: yes	1.80	1.44–2.25	<0.0001	2.00	1.59–2.54	<0.0001
Main caregiver: yes	1.13	0.90–1.43	0.28			
Formal help: yes	1.48	1.15–1.91	0.003			
Informal help: yes	0.80	0.58–1.11	0.17			
Dependence for the ADLs: yes	2.17	1.55–3.05	<0.0001	1.81	1.24–2.64	0.002
Walking difficulties: yes	1.40	1.05–1.88	0.02			
Balance difficulties: yes	1.60	1.29–2.00	<0.0001	1.37	1.09–1.73	0.007
Dementia syndrome: yes	2.03	1.64–2.52	<0.0001	1.80	1.42–2.29	<0.0001
Delirium: yes	1.03	0.77–1.37	0.86			
Depression or risk thereof: yes	1.29	1.04–1.60	0.02			
Serum albumin level: <35 g/L	0.94	0.76–1.16	0.55			
Total MNA score: <24	1.11	0.89–1.38	0.36			
Risk of pressure ulcers: yes	1.78	1.43–2.22	<0.0001	1.42	1.10–1.82	0.006
Visual impairment: yes	1.19	0.96–1.48	0.12			
Hearing impairment: yes	1.15	0.93–1.43	0.20			
Recent hospital admission: yes	1.01	0.79–1.29	0.94			
Day of hospital admission: weekend	1.24	0.97–1.58	0.08			

## Data Availability

Data can be shared on reasonable request at moustapha.drame@chu-martinique.fr.
